# VCO_2_ calorimetry: stop tossing stones, it’s time for building!

**DOI:** 10.1186/s13054-016-1575-z

**Published:** 2016-12-16

**Authors:** Elisabeth De Waele, Patrick M. Honoré, Herbert D. Spapen

**Affiliations:** ICU Department, Universitair Ziekenhuis Brussel, Vrije Universiteit Brussel, 101 Laarbeeklaan, 1090 Brussels, Belgium

**Keywords:** Energy expenditure, Nutrition, Intensive care unit

We followed with interest the discussion [1, 2] fueled by the study of Stapel et al. [3] who reported fairly accurate assessment of energy expenditure (EE) in critically ill patients based on ventilator-derived carbon dioxide production (VCO_2_). The proposed technique is elegant and valid but has inherent limitations. It is applicable in patients who are in one way or another ventilator-dependent but not in spontaneously breathing yet oxygen-dependent subjects. We concur that VO_2_ is the most relevant variable for EE measurement. However, the most accurate and precise estimation of EE in a critically ill population can only be obtained by sampling of inspired and expired oxygen/carbon dioxide concentrations and measuring expired gas flow. This is the core task of indirect calorimetry [4].

Initiative has been undertaken to develop a ‘full option’, easy-to-use, accurate, and affordable indirect calorimeter. The project is supported by the European Society of Intensive Care Medicine and the European Society of Parenteral and Enteral Nutrition [5] and has actually reached Technology Readiness Level. It is probably only a matter of time before such a device will render all current mathematical uproar obsolete.

## Abbreviations

EE: Energy expenditure
VCO_2_: Carbon dioxide production
